# Met and Unmet Breastfeeding Goals Following Full-Term Deliveries

**DOI:** 10.1001/jamanetworkopen.2026.32783

**Published:** 2026-09-08

**Authors:** Deanna Nardella, Veronika Shabanova, Mona Sharifi, Sarah N. Taylor

**Affiliations:** 1 Department of Pediatrics, Yale School of Medicine, New Haven, Connecticut

## Abstract

This cross-sectional study examines the prevalence of met and unmet prenatal breastfeeding goals and describes and compares individuals with or without these outcomes in a sample of adults who gave birth at full term.

## Introduction

A paucity of person-centered outcomes (PCOs) exists in the breastfeeding literature.^1^ Adopting PCOs will be critical to move beyond breastfeeding exclusivity and duration as sole measures of perceived breastfeeding success and address gaps of how breastfeeding interventions impact maternal emotional well-being.^2^ One PCO to consider is whether one meets their breastfeeding goal. National analyses of 2005 to 2007 and 2013 data from the Centers from Disease Control and Prevention identified that more than one-half^1,3^ and up to three-quarters^4^ of individuals ceased breastfeeding earlier than intended or desired. Updated analyses are needed, as several national policies have expanded access to breastfeeding support professionals, technologies, and workplace protections since these studies.^1^ To assess our progress and inform future PCOs, we aimed to quantify the prevalence of any, unmet, and met prenatal breastfeeding goals, and to describe and compare individuals with and without these outcomes in a US sample.

## Methods

We present secondary analyses of cross-sectional electronic survey data from adults who delivered a full-term infant in the US in the prior 3 to 24 months, reported any pump use after delivery, and had proficiency in English or Spanish. The Yale Institutional Review Board (study 2000039769) determined this study to be exempt from annual review. The AAPOR reporting guidelines informed our manuscript structure. Using methods detailed in our prior publication,^5^ we commissioned a survey panel provider (XandY) to survey individuals on their pump use following full-term deliveries. Recruitment occurred between January 15 and 30, 2026, and was targeted to match select sample demographics related to breastfeeding outcomes (ie, Medicaid, education, non-Hispanic Black and White race, and Hispanic ethnicity) with those reported in a 2023 national analysis of breastfeeding outcomes.^6^

Questions adapted from national Centers for Disease Control and Prevention surveys (Infant Feeding Practices Survey-II, National Immunization Survey, and Pregnancy Risk and Assessment Monitoring System)^6,7,8^ assessed sociodemographic and health covariates known to be associated with breastfeeding. All data, including race and ethnicity, were self-reported via the survey instrument. Measures for breastfeeding satisfaction and success were adapted from DeYoreo et al.^9^ To measure the (1) presence of a prenatal breastfeeding goal and (2) whether this goal was unmet or met, we asked (1) did the participant have a prenatal plan to try breastfeeding (yes or no), (2) did they have a goal for how long they wanted to feed breast milk (yes or no), and (3) did they reach this goal (yes; no, but I feel okay with this; no, and I feel disappointed by this; I am still working toward my goal; or unsure). Six breastfeeding and lactation medicine experts and 4 community partners who serve young families reviewed the survey to improve content validity. Cognitive interviews were performed with 8 birthing individuals. Descriptive summaries of variables of interest are presented by (1) the presence of a prenatal breastfeeding goal and (2) whether this goal was unmet or met. Between-group comparisons were done via χ^2^ tests (type I error, .05), and associations were summarized with unadjusted odds ratios and 95% CIs. Data were analyzed with Stata statistical software version 17.0 (StataCorp).

## Results

Of 1437 individuals screened, 928 were eligible, 719 provided valid responses, and 7 were excluded for completing the survey under one-third of the median completion time (9 minutes, 43 seconds). Our final sample included 712 participants (77% response rate) with a mean (SD) age of 31.7 (6.2) years and from 48 US states. Mean (SD) infant age at survey completion was 13.0 (6.5) months (Table 1).

**Table 1.** Characteristics of Individuals From 48 US States Who Delivered a Full-Term Infant Between 2024 and 2025 by the Presence of a Prenatal Breastfeeding Goal^a^

Characteristic	Participants, No. (%)			χ^2^ *P* value
	Total (N = 712)	No prenatal goal (n = 121 [16.99%])	Prenatal goal (n = 591 [83.01%])	
Participant age, y				
18-24	86 (12.08)	14 (11.57)	72 (12.18)	.97
25-30	246 (34.55)	39 (32.23)	207 (35.03)	
31-35	192 (26.97)	35 (28.93)	157 (26.57)	
36-40	121 (16.99)	21 (17.36)	100 (16.92)	
≥41	67 (9.41)	12 (9.92)	55 (9.31)	
Infant age, mo				
3-6	148 (20.79)	28 (23.14)	120 (20.30)	.88
7-12	223 (31.32)	35 (28.93)	188 (31.81)	
13-18	161 (22.61)	27 (22.31)	134 (22.67)	
19-24	180 (25.28)	31 (25.62)	149 (25.21)	
Infant sex				
Female	439 (61.66)	70 (57.85)	369 (62.44)	.48
Male	263 (36.94)	48 (39.67)	215 (36.38)	
Nonresponse	10 (1.12)	3 (2.48)	7 (1.18)	
Infant delivery hospitalization				
0-2 d	419 (58.85)	77 (63.64)	342 (57.87)	.63
3-5 d	238 (33.43)	33 (27.27)	205 (34.69)	
6-14 d	39 (5.48)	7 (5.79)	32 (5.41)	
>2 wk	7 (0.98)	2 (1.65)	5 (0.85)	
Infant not born in hospital	8 (1.12)	2 (1.65)	6 (1.02)	
Nonresponse	1 (0.14)	0	1 (0.17)	
Delivery mode				
Vaginal	527 (74.02)	84 (69.42)	443 (74.96)	.39
Cesarean delivery	184 (25.84)	37 (30.58)	147 (24.87)	
Nonresponse	1 (0.14)	0	1 (0.17)	
Primipara	238 (33.43)	41 (33.88)	197 (33.33)	.91
Prior breastfeeding experience	446 (62.64)	73 (60.33)	373 (63.11)	.56
Maternal diagnoses before or after delivery^b^				
Anxiety	308 (43.26)	63 (52.07)	245 (41.46)	.03
Depression	253 (35.53)	48 (39.67)	205 (34.69)	.30
Diabetes (gestational, type 1, or type 2)	69 (9.69)	13 (10.74)	56 (9.48)	.67
High blood pressure	151 (21.21)	27 (22.31)	124 (20.98)	.74
None of the above	263 (36.94)	39 (32.23)	224 (37.90)	.24
Self-reported race^b^				
American Indian or Alaska Native	8 (1.12)	2 (1.65)	6 (1.02)	.90
Asian^c^	29 (4.07)	3 (2.48)	26 (4.40)	
Black or African American	104 (14.61)	16 (13.22)	88 (14.89)	
Hispanic, no race reported	70 (9.83)	14 (11.57)	56 (9.48)	
Middle Eastern or North African	4 (0.56)	1 (0.83)	3 (0.51)	
Native Hawaiian or Pacific Islander	2 (0.28)	0	2 (0.34)	
White	434 (60.96)	72 (59.50)	362 (61.25)	
>1 Race^d^	38 (5.34)	7 (5.79)	31 (5.25)	
Other^e^	14 (1.97)	4 (3.31)	10 (1.69)	
Prefer not to answer	9 (1.26)	2 (1.65)	7 (1.18)	
Self-reported Hispanic ethnicity	127 (17.84)	22 (18.18)	105 (17.77)	.91
Survey language				
English	652 (91.57)	107 (88.43)	545 (92.22)	.17
Spanish	60 (8.43)	14 (11.57)	46 (7.78)	
Health literacy^f^				
Limited	8 (1.12)	0	8 (1.35)	.19
Marginal	54 (7.58)	13 (10.74)	41 (6.94)	
Adequate	644 (90.45)	106 (87.60)	538 (91.03)	
Nonresponse	6 (0.84)	2 (1.65)	4 (0.68)	
Highest formal education				
High school or less	269 (37.78)	66 (54.54)	203 (34.35)	.001
Associate’s degree	112 (15.73)	17 (14.05)	95 (16.07)	
Bachelor’s degree	223 (31.32)	27 (22.31)	196 (33.16)	
Master’s or doctoral degree or higher	102 (14.33)	9 (7.44)	93 (15.74)	
Nonresponse	6 (0.84)	2 (1.65)	4 (0.68)	
Insurance at time of delivery				
Private (employer, parent, spouse, purchased independently)	371 (52.11)	48 (39.67)	323 (54.65)	.04
Medicaid	285 (40.03)	62 (51.24)	223 (37.73)	
Other^g^	30 (4.21)	6 (4.96)	24 (4.06)	
No insurance, self-pay	20 (2.81)	3 (2.48)	17 (2.88)	
Nonresponse	6 (0.84)	2 (1.65)	4 (0.68)	
Employed during pregnancy	458 (64.33)	73 (60.33)	385 (65.14)	.31
US region				
Midwest	156 (21.91)	24 (19.83)	132 (22.34)	.04^h^
Northeast	132 (18.54)	21 (17.36)	111 (18.78)	
South and District of Columbia	281 (39.47)	48 (39.67)	233 (39.42)	
West	125 (17.56)	20 (16.53)	105 (17.77)	
Nonresponse	18 (2.53)	8 (6.61)	19 (1.69)	

^a^ Participants gave birth in 48 US states (all but New Hampshire and North Dakota) and the District of Columbia.

^b^ Participants were able to select more than 1 response.

^c^ Asian race includes Chinese, Filipino, Indian, Japanese, Korean, Vietnamese, and Japanese or Korean mixed.

^d^ More than 1 race includes American Indian or Alaska Native, Black or African American, Chinese, Filipino, Indian, Japanese, Korean, White, and Vietnamese.

^e^ Participants selected the response option other for the question asking their racial identity.

^f^ Health literacy categories are based on the answer to single validated question, “How confident are you filling out medical forms by yourself?” with responses of not at all or a little bit classified as limited, somewhat classified as marginal, and quite a bit or extremely classified as adequate.

^g^ Other insurance includes Indian Health Service (n = 4), Military or TRICARE (n = 8), other insurance (n = 17), and unsure (n = 1).

^h^ Differences observed between US region are not considered meaningful as representation from region is comparable between those with and without a prenatal goal. The significant *P* value is attributed to the low nonresponse rate, not the geographic location category.

Almost all participants planned to try breastfeeding (99.2%; 95% CI, 98.1%-99.6%), most of whom (83.7%; 95% CI, 80.8%-86.3%) had a prenatal goal for how long they wanted to feed breast milk. Participants with vs without prenatal goals did not differ significantly by age, race, ethnicity, gravida, or employment status. Among the 474 participants who had ceased breastfeeding by survey completion, 59.7% (95% CI, 55.2%-64.0%) reported met and 40.3% (95% CI, 36.0%-44.8%) reported unmet goals. Most participants with unmet goals (116 participants [60.7%]) reported disappointment with this outcome and had 8- to 10-fold unadjusted odds of strongly disagreeing that they felt satisfied with and/or successful at breastfeeding. Those with unmet vs met goals had significantly higher odds of reporting anxiety, depression, diabetes, hypertension, breastfeeding challenges, not receiving helpful breastfeeding support, Medicaid insurance, lower formal education, and less family work leave (Table 2).

**Table 2.** Characteristics of Individuals From 45 US States Who Delivered a Full-Term Infant From 2024 to 2025 by Unmet and Met Prenatal Breastfeeding Goals^a^

Characteristic	Participants, No. (%)			Unmet goals, unadjusted OR (95% CI)
	Total (n = 474)^b^	Unmet goal (n = 191 [40.30%])	Met goal (n = 283 [59.70%])	
Participant age, y				
18-24	59 (12.45)	25 (13.09)	34 (12.01)	1.04 (0.57-1.91)
25-30	162 (34.18)	67 (35.08)	95 (33.57)	1 [Reference]
31-35	123 (25.95)	47 (24.61)	76 (26.86)	0.88 (0.54-1.42)
36-40	83 (17.51)	37 (19.37)	46 (16.25)	1.14 (0.67-1.95)
≥41	47 (9.92)	15 (7.85)	32 (11.31)	0.67 (0.33-1.32)
Infant age at time of survey, mo				
3-6	73 (15.40)	30 (15.71)	43 (15.19)	1.07 (0.60-1.90)
7-12	142 (29.96)	56 (29.32)	86 (30.39)	1 [Reference]
13-18	122 (25.74)	52 (27.23)	70 (24.73)	1.14 (0.70-1.87)
19-24	137 (28.90)	53 (27.75)	84 (29.68)	0.97 (0.60-1.57)
Infant sex				
Female	298 (62.87)	118 (61.78)	180 (63.6)	1 [Reference]
Male	170 (35.86)	72 (37.70)	98 (34.63)	1.12 (0.76-1.64)
Nonresponse	6 (1.27)	1 (0.52)	5 (1.77)	NA^c^
Infant delivery hospitalization				
0-2 d	272 (57.38)	101 (52.88)	171 (60.42)	1 [Reference]
3-5 d	162 (34.18)	74 (38.74)	88 (31.10)	1.42 (0.96-2.11)
6-14 d	29 (6.12)	11 (5.76)	18 (6.36)	1.04 (0.47-2.28)
>2 wk	5 (1.05)	4 (2.09)	1 (0.35)	6.77 (0.75-61.43)
Infant not born in hospital	5 (1.05)	1 (0.52)	4 (1.41)	0.42 (0.05-3.84)
Nonresponse	1 (0.21)	0	1 (0.35)	NA^c^
Delivery mode				
Vaginal	350 (73.84)	132 (69.11)	218 (77.03)	1 [Reference]
Cesarean delivery	123 (25.95)	59 (30.89)	64 (22.61)	1.52 (1.01-2.31)
Nonresponse	1 (0.21)	0	1 (0.35)	NA^c^
Primipara	157 (33.12)	69 (36.13)	88 (31.10)	1.25 (0.85-1.85)
Had prior breastfeeding experience	299 (63.08)	111 (58.12)	188 (66.43)	0.70 (0.48-1.02)
Self-reported maternal diagnoses before or after pregnancy^d^				
Anxiety	206 (43.46)	96 (50.26)	110 (38.87)	1.59 (1.10-2.30)
Depression	177 (37.34)	87 (45.55)	90 (31.80)	1.79 (1.23-2.62)
Diabetes (gestational, type 1, or type 2)	50 (10.55)	27 (14.14)	23 (8.13)	1.86 (1.03-3.36)
High blood pressure	109 (23.00)	54 (28.27)	55 (19.43)	1.63 (1.06-2.52)
None of the above	164 (34.60)	48 (25.13)	116 (40.99)	0.48 (0.32-0.72)
Breastfeeding challenges^d^				
Pain or discomfort	295 (62.24)	109 (57.07)	186 (65.72)	0.69 (0.48-1.01)
Latch difficulties	209 (44.09)	96 (50.26)	113 (39.93)	1.52 (1.05-2.20)
Low milk production	185 (39.03)	114 (59.69)	71 (25.09)	4.42 (2.98-6.56)
Cracked or blistered nipple(s)	160 (33.76)	65 (34.03)	95 (33.57)	1.02 (0.69-1.51)
Plugged ducts	96 (20.25)	34 (17.80)	62 (21.91)	0.77 (0.49-1.23)
Milk overproduction (ie, oversupply)	88 (18.57)	19 (9.95)	69 (24.38)	0.34 (0.20-0.59)
Infection breast (eg, mastitis)	28 (5.91)	8 (4.19)	20 (7.07)	0.58 (0.25-1.33)
Had none of the above challenges	43 (9.07)	5 (2.62)	38 (13.43)	0.17 (0.07-0.45)
Needed help with breastfeeding				
Yes, and I got the help I needed	261 (55.06)	90 (47.12)	171 (60.42)	0.85 (0.56-1.30)
Yes, but I did not get help or the help I received was not useful	68 (14.35)	46 (24.08)	22 (7.77)	3.38 (1.84-6.22)
No, I did not need help with breastfeeding	144 (30.38)	55 (28.80)	89 (31.45)	1 [Reference]
Unsure	1 (0.21)	0	1 (0.35)	NA
In general, I am satisfied with my breastfeeding experience				
Strongly agree	201 (42.41)	28 (14.66)	173 (61.13)	0.09 (0.04-0.20)
Somewhat agree	142 (29.96)	58 (30.37)	84 (29.68)	0.39 (0.19-0.81)
Neither agree nor disagree	39 (8.23)	25 (13.09)	14 (4.95)	1 [Reference]
Somewhat disagree	61 (12.87)	51 (26.70)	10 (3.53)	2.86 (1.11-7.33)
Strongly disagree	31 (6.54)	29 (15.18)	2 (0.71)	8.12 (1.68-39.23)
In general, I feel successful at breastfeeding				
Strongly agree	209 (44.09)	25 (13.09)	184 (65.02)	0.06 (0.03-0.15)
Somewhat agree	129 (27.22)	49 (25.65)	80 (28.27)	0.28 (0.12-0.65)
Neither agree nor disagree	29 (6.12)	20 (10.47)	9 (3.18)	1 [Reference]
Somewhat disagree	62 (13.08)	54 (28.27)	8 (2.83)	3.04 (1.03-8.96)
Strongly disagree	45 (9.49)	43 (22.51)	2 (0.71)	9.68 (1.91-48.96)
Participant work leave duration^e^				
<6 wk	72 (23.6)	28 (24.78)	44 (22.92)	1.91 (0.89-4.12)
7-12 wk	111 (36.39)	39 (34.51)	72 (37.50)	1.63 (0.79-3.34)
4-6 mo	56 (18.36)	14 (12.39)	42 (21.88)	1 [Reference]
>6 mo	45 (14.75)	19 (16.81)	26 (13.54)	2.19 (0.94-5.11)
I do not plan to return to work	21 (6.89)	13 (11.5)	8 (4.17)	4.88 (1.68-14.19)
Other caregiver work leave				
Did not take work leave	117 (24.68)	57 (29.84)	60 (21.20)	1.81 (1.17-2.79)
Took work leave	302 (63.71)	104 (54.45)	198 (69.96)	1 [Reference]
Does not apply to my family	51 (10.76)	29 (15.18)	22 (7.77)	2.51 (1.37-4.59)
Nonresponse	4 (0.84)	1 (0.52)	3 (1.06)	NA^c^
Self-reported race^d^				
American Indian or Alaska Native	6 (1.27)	3 (1.57)	3 (1.06)	NA
Asian^f^	21 (4.43)	8 (4.19)	13 (4.59)	0.86 (0.35-2.14)
Black or African American	74 (15.61)	24 (12.57)	50 (17.67)	0.67 (0.39-1.15)
Hispanic, no race reported	46 (9.70)	17 (8.90)	29 (10.25)	0.82 (0.43-1.56)
Middle Eastern or North African	3 (0.63)	1 (0.52)	2 (0.71)	0.70 (0.06-7.80)
Native Hawaiian or Pacific Islander	1 (0.21)	0	1 (0.35)	NA
White	283 (59.70)	118 (61.78)	165 (58.30)	1 [Reference]
>1 Race^g^	28 (5.91)	16 (8.38)	12 (4.24)	1.86 (0.85-4.09)
Other^h^	7 (1.48)	3 (1.57)	4 (1.41)	0.987 (0.36-2.65)^g^
Prefer not to answer	5 (1.05)	1 (0.52)	4 (1.41)	
Self-reported Hispanic ethnicity	86 (18.14)	31 (16.23)	55 (19.43)	0.80 (0.50-1.30)
Survey language				
English	434 (91.56)	177 (92.67)	257 (90.81)	1 [Reference]
Spanish	40 (8.44)	14 (7.33)	26 (9.19)	0.78 (0.40-1.54)
Health literacy^i^				
Limited health literacy	8 (1.69)	4 (2.09)	4 (1.41)	1.50 (0.37-6.09)
Marginal health literacy	29 (6.12)	13 (6.81)	16 (5.65)	1.22 (0.57-2.60)
Adequate health literacy	433 (91.35)	173 (90.58)	260 (91.87)	1 [Reference]
Nonresponse	4 (0.84)	1 (0.52)	3 (1.06)	NA^c^
Less than a college degree	256 (54.01)	126 (65.97)	130 (45.94)	2.27 (1.55-3.33)
Primary insurance at delivery				
Private (employer, parent, spouse, purchased independently)	249 (52.53)	82 (42.93)	167 (59.01)	1 [Reference]
Medicaid	190 (40.08)	95 (49.74)	95 (33.57)	2.04 (1.38-3.00)
Other^j^	18 (3.80)	6 (3.14)	12 (4.24)	1.02 (0.37-2.81)
No insurance, self-pay	13 (2.74)	7 (3.66)	6 (2.12)	2.38 (0.77-7.30)
Nonresponse	4 (0.84)	1 (0.52)	3 (1.06)	NA^c^
US region^a^				
Midwest	105 (22.15)	50 (26.18)	55 (19.43)	1.51 (0.84-2.70)
Northeast	85 (17.93)	32 (16.75)	53 (18.73)	1 [Reference]
South and District of Columbia	190 (40.08)	74 (38.74)	116 (40.99)	1.06 (0.62-1.79)
West	85 (17.93)	32 (16.75)	53 (18.73)	1.00 (0.54-1.86)
Nonresponse	9 (1.90)	3 (1.57)	6 (2.12)	NA^c^

Abbreviations: NA, not applicable; OR, odds ratio.

^a^ Participants gave birth in 45 US states and the District of Columbia. States not represented included Delaware, Maine, Montana, New Hampshire, and North Dakota.

^b^ Of 591 participants with a prenatal goal for how long they wanted to feed breast milk, 117 were excluded as they were still working toward their goal at survey completion.

^c^ Not estimated due to low count and low generalizability.

^d^ Participants were able to select more than 1 response.

^e^ Includes 305 participants who reported (1) working during pregnancy and (2) an unmet or met prenatal breastfeeding goal.

^f^ Asian race includes Chinese, Filipino, Indian, Japanese, Korean, Vietnamese, and Japanese or Korean mixed.

^g^ More than 1 race includes American Indian or Alaska Native, Black or African American, Chinese, Filipino, Indian, Japanese, Korean, White, and Vietnamese.

^h^ Prevalences reported for other race reflect participants who selected other for their racial identity. The unadjusted OR for other race includes participants who selected American Indian or Alaska Native, Middle Eastern or North African, Native Hawaiian or Pacific Islander, other, and prefer not to answer. The latter subgroups were merged due to their small sample size.

^i^ Health literacy categories are based on the answer to a single validated question, “How confident are you filling out medical forms by yourself?” with responses of not at all or a little bit classified as limited, somewhat classified as marginal, and quite a bit or extremely classified as adequate.

^j^ Other insurance includes Indian Health Service (n = 2), Military or TRICARE (n = 4), other insurance (n = 11), and unsure (n = 1).

## Discussion

To our knowledge, this cross-sectional study is the first national analysis of unmet and met prenatal breastfeeding goals following US full-term deliveries in the prior decade.^3,4^ Although the trends have improved from prior analyses,^1,3^ we identified a high prevalence of unmet goals in our national sample (40.3%). Those with unmet goals had higher odds of maternal comorbidities, breastfeeding challenges, and not receiving helpful breastfeeding support when needed.

Our findings are directly generalizable to individuals with a prenatal intent to try breastfeeding and any postpartum pump use, representing up to 90% of US breastfeeding individuals.^10^ Sampling bias is a risk when recruiting individuals who previously agreed to be approached for online surveys. Nonetheless, using a survey panel provider allowed for our geographically and demographically diverse national sample, which improves the generalizability of our findings. In addition, although individuals’ perceptions of unmet or met goals may change over time, we observed no difference in infant age at survey completion between those with met vs unmet goals. Moreover, our findings can inform studies to develop validated PCOs to advance knowledge of how breastfeeding interventions impact maternal well-being and support more families in meeting their infant feeding goals.^2^

## Notes

Supplement. Data Sharing Statement

## References

[1] Young M, Caswell JA, Asiodu I, eds. National Academies of Sciences, Engineering, and Medicine; Division of Behavioral and Social Sciences and Education; Board on Behavioral, Cognitive, and Sensory Sciences; Board on Children, Youth, and Families; Committee on Understanding Breastfeeding Promotion, Initiation and Support Across the United States. Breastfeeding in the United States: Strategies to Support Families and Achieve National Goals. National Academies Press; 2025. Accessed May 26, 2026. https://www.ncbi.nlm.nih.gov/books/NBK619950/41468480

[2] US Preventive Services Task Force. Primary care behavioral counseling interventions to support breastfeeding: US Preventive Services Task Force recommendation statement. JAMA. 2025;333(17):1520-1526. doi:10.1001/jama.2025.3650 40198087

[3] Gregory EF, Butz AM, Ghazarian SR, Gross SM, Johnson SB. Met expectations and satisfaction with duration: a patient-centered evaluation of breastfeeding outcomes in the Infant Feeding Practices Study II. J Hum Lact. 2015;31(3):444-451. doi:10.1177/0890334415579655 25858883

[4] Hamner HC, Beauregard JL, Li R, Nelson JM, Perrine CG. Meeting breastfeeding intentions differ by race/ethnicity: Infant and Toddler Feeding Practices Study-2. Matern Child Nutr. 2021;17(2):e13093. doi:10.1111/mcn.13093 33006242 PMC7988881

[5] Nardella D, Damian K, Glassman ME, Bassler B, Taylor SN, Silpe J. A national survey: trends in U.S. postpartum pumping practices after term deliveries. Breastfeed Med. Published online November 10, 2025. doi:10.1177/15568253251393228 PMC13435351 41204728

[6] Diaz LE, Yee LM, Feinglass J. Rates of breastfeeding initiation and duration in the United States: data insights from the 2016-2019 Pregnancy Risk Assessment Monitoring System. Front Public Health. 2023;11:1256432. doi:10.3389/fpubh.2023.1256432 38192551 PMC10773697

[7] O’Sullivan EJ, Geraghty SR, Cassano PA, Rasmussen KM. Comparing alternative breast milk feeding questions to U.S. breastfeeding surveillance questions. Breastfeed Med. 2019;14(5):347-353. doi:10.1089/bfm.2018.0256 30939039

[8] Keim SA, Smith K, Ingol T, Li R, Boone KM, Oza-Frank R. Improved estimation of breastfeeding rates using a novel breastfeeding and milk expression survey. Breastfeed Med. 2019;14(7):499-507. doi:10.1089/bfm.2018.0258 31509466 PMC6909394

[9] DeYoreo M, Kapinos K, Waymouth M, James KF, Demirci J, Uscher-Pines L. The impact of telelactation on breastfeeding satisfaction at 6 months postpartum: evidence from a randomized controlled trial. Acad Pediatr. 2025;25(7):102837. doi:10.1016/j.acap.2025.102837 40253004 PMC12400814

[10] Nardella D, Canavan M, Sharifi M, Taylor S. Quantifying the association between pump use and breastfeeding duration. J Pediatr. 2024;274:114192. doi:10.1016/j.jpeds.2024.114192 39004167 PMC11499033

