# Supplementary material for: Met and Unmet Breastfeeding Goals Following Full-Term Deliveries
Source: JAMA Netw Open. 2026 Sep 8;9(9):e2632783. doi: 10.1001/jamanetworkopen.2026.32783 (PMC13555374; doi:10.1001/jamanetworkopen.2026.32783)
Supplement: Supplementary file 1 [file jamanetwopen-e2632783-s001.pdf]

Data Sharing Statement

Nardella. Met and Unmet Breastfeeding Goals Following Full-Term Deliveries. *JAMA Netw Open*. Published September 08, 2026. doi:10.1001/jamanetworkopen.2026.32783

Data
Data available: Yes
Data types: Deidentified participant data
How to access data: Interested individuals can contact breastfeedingresearch@yale.edu for data inquiries.
When available: With publication

Supporting Documents
Document types: None

Additional Information
Who can access the data: Researchers whose proposed use of the data has been approved.
Types of analyses: For any purpose
Mechanisms of data availability: With a signed data access agreement
